# Supplementary material for: Navigating uncertain illness trajectories for young children with serious infectious illness: a modified grounded theory study
Source: BMC Health Serv Res. 2022 Aug 30;22:1103. doi: 10.1186/s12913-022-08420-5 (PMC9427158; doi:10.1186/s12913-022-08420-5)
Supplement: Supplementary file 2 — Additional file 2: S2 Fig.Table 1. Stage 1 Characteristics of parent/carer participants and their affected child (N = 22~). Table 2. Stage 1 Characteristics of Health professional (HP) participants (N = 14~). Table 3. Stage 2 Characteristics of parent participants (N = 18~). Table 4. Stage 2 Characteristics of health professional (HP) participants (N = 16~). Table 5. Stage 1 Characteristics of each family and affected child. Table 6. Stage 2 Characteristics of each family and affected child. Table 7. Stage1 Illness trajectories. Table 8. Stage 2 Illness trajectories [file 12913_2022_8420_MOESM2_ESM.docx]

**S2 Fig Tables**

**Table 1 Stage 1 Characteristics of parent/carer participants and their affected child (N=22~)**

| Characteristic | Number of parents (%) |  | Characteristic | Number of parents (%) |
| --- | --- | --- | --- | --- |
| Age | |  | **Relationship to the child** | |
| 25-29 years | 3 (13%) |  | Parent: Mother  Parent: Father  Other family carer | 11 (50%)  8 (36%)  3 (14%) |
| 30-39 years | 10 (44%) |  |  |  |
| 40-49 years | 0 |  |  |  |
| 50-59 years | 1 (4%) |  |  |  |
| 60+ years | 3 (13%) |  |  |  |
|  |  |  |  |  |
| Gender | |  | **Income** | |
| Female | 12 (52%) |  | Less than 10,000 | 3 (13%) |
| Male | 9 (39%) |  | 10,000-19,999 | 5 (22%) |
| Ethnicity | |  | 20,000-29,999 | 4 (17%) |
| White British | 12 (52%) |  | 30,000-39,999 | 5 (22%) |
| Indian | 6 (26%) |  | 40,000-49,999 | 0 |
| Employment | |  | 50,000-59,999 | 2 (9%) |
| Employed (part or full time) | 8 (35%) |  | 60,000-79,999 | 2 (9%) |
| Unemployed or retired | 3 (13%) |  | 80,000-99,999 | 1 (4%) |
| Caring for family at home | 5 (22%) |  | 100,000+ | 3 (13%) |
| Age of affected child* | |  | **Diagnoses of affected child*&**** | |
| Under 6 months | 1 (8%) |  | Acute Respiratory | 12 (52%) |
| 6-12 months | 2 (17%) |  | Acute exacerbation of recurrent respiratory | 5 (22%) |
| 13-23 months | 2 (17%) |  |  |  |
| 2-4 years old | 7 (58%) |  | Acute disseminated encephalomyelitis (ADEM) | 1 (4%) |
|  |  |  |  |  |
|  |  |  | Tonsillitis | 1 (4%) |
|  |  |  | Sepsis and Septicaemia | 2 (9%) |
| ~Although 22 parents/carers completed the questionnaire, questions were not compulsory and therefore each question was not always completed by 100% of parents.  *Based on the number of families (N=12) engaged in Stage 1, not on the total number of parents (N=22) participating in Stage 1.  **Many children had multiple diagnoses. | | | | |

**Table 2 Stage 1 Characteristics of Health professional (HP) participants (N=14~)**

| Characteristic | Number of HPs (%) |  | Characteristic | Number of HPs (%) |
| --- | --- | --- | --- | --- |
| Age | |  | **Service type**** | |
| 21-29 years | 5 (36%) |  | Ambulance Service | 6 (43%), |
| 30-39 years | 5 (36%) |  | Emergency Care | 8 (57%) |
| 40-49 years | 1 (7%) |  | Other*** | 2 (14%) |
| 50-59 years | 3 (21%) |  | **Job title** | |
| Gender | |  | Emergency Medical Technician | 3 (21%) |
| Female | 9 (64%) |  | Emergency Medical Dispatcher | 1 (7%) |
| Male | 4 (29%) |  | Emergency Medical Consultant | 1 (7%) |
| Ethnicity | |  | Emergency Care Assistant | 1 (7%) |
| White British | 11 (79%) |  | Emergency Care Nurse | 2 (14%) |
| Indian | 1 (7%) |  | Junior Doctor | 1 (7%) |
| Other* | 2 (14%) |  | Paramedic | 3 (21%) |
| Employment | |  | Health Advisor | 2 (14%) |
| Employed (full time) | 14 (100%) |  |  |  |
| ~Although 14 health professionals completed the questionnaire, questions were not compulsory and therefore each question was not always completed by 100% of professionals.  *Welsh, White other unspecified  **Some staff work across multiple services  ***Emergency Service - Air Ambulance, Paediatric Ward | | | | |

**Table 3 Stage 2 Characteristics of parent participants (N=18~)**

| Characteristic | Number of parents (%) |  | Characteristic | Number of parents (%) |
| --- | --- | --- | --- | --- |
| Age | |  | **Relationship to the child** | |
| 30-39 years | 11 (61%) |  | Parent: Mother | 15 (83%), |
| 40-49 years | 5 (28%) |  | Parent: Father | 2 (11%) |
| Gender | |  | **Income** | |
| Female | 14 (78%) |  | Less than 10,000 | 2 (11%) |
| Male | 2 (11%) |  | 10,000-19,999 | 3 (17%) |
| Ethnicity | |  | 20,000-29,999 | 0 |
| White British | 12 (67%) |  | 30,000-39,999 | 0 |
| White other* | 3 (17%) |  | 40,000-49,999 | 1 (6%) |
| Employment Status | |  | 50,000-59,999 | 1 (6%) |
| Employed (part or full time) | 12 (67%) |  | 60,000-79,999 | 3 (17%) |
| Unemployed | 1 (6%) |  | 80,000-99,999 | 4 (22%) |
| Caring for family at home | 3 (17%) |  | 100,000+ | 2 (17%) |
| Age of affected child** | |  | **Diagnoses of affected child**&***** | |
| Under 6 months | 6 (38%) |  | Acute Respiratory | 1 (6%) |
| 6-12 months | 4 (25%) |  |  |  |
| 13-23 months | 2 (12%) |  | Sepsis and Septicaemia | 6 (38%) |
| 2-4 years old | 4 (25%) |  |  |  |
|  |  |  | Meningitis | 14 (88%) |
|  |  |  |  |  |
| ~Although 18 parents completed the questionnaire, questions were not compulsory and therefore each question was not always completed by 100% of parents.  *European, Scottish, Other unspecified.  **Based on the number of families (N=16) engaged in Stage 2, not on the total number of parents (N=18) engaged in Stage 2.  ***Many children have multiple diagnoses. | | | | |

**Table 4 Stage 2 Characteristics of health professional (HP) participants (N=16~)**

| Characteristic | Number of HPs (%) |  | Characteristic | Number of HPs (%) |
| --- | --- | --- | --- | --- |
| Age | |  | **Service type** | |
| 21-29 years | 2 (13%) |  | General Practice | 5 (32%) |
| 30-39 years | 6 (38%) |  | Emergency Care | 5 (32%) |
| 40-49 years | 4 (25%) |  | Ambulance Service | 2 (13%) |
| 50-59 years | 4 (25%) |  | Other** | 4 (25%) |
| Gender | |  | **Job title** | |
| Female | 9 (56%) |  | General Practitioner | 5 (32%) |
| Male | 5 (32%) |  | Paediatric Emergency Medical Consultant | 4 (25%) |
| Ethnicity | |  |  |  |
| White British | 10 (63%) |  | Emergency Care Children’s Nurse | 1 (6%) |
| South Asian* | 3 (19%) |  |  |  |
| African | 1 (6%) |  | Community Children’s Nurse | 1 (6%) |
| Other* | 2 (13%) |  |  |  |
| Employment | |  | Paramedic | 2 (13%) |
| Employed (full time) | 12 (75%) |  | Other*** | 3 (19%) |
| Employed (part time) | 4 (25%) |  |  |  |
| ~Although 16 health professionals completed the questionnaire, questions were not compulsory and therefore each question was not always completed by 100% of professionals.  *Indian, Pakistani, Bangladeshi  ** NHS111, Community  ***Community Pharmacist, Dental Hygienist Oral Health Lead, Health Advisor | | | | |

**Table 5 Stage 1 Characteristics of each family and affected child**

TH Teaching hospital; DGH District general hospital; NP Nurse Practitioner; CAU Child Assessment Unit.

# Table 6 Stage 2 Characteristics of each family and affected child

FG1 = Parent Focus group 1, August 2019; FG2 = Parent Focus group 2, October 2019; FG3 = Parent Focus group 3, October 2019; T = Parent Focus group alternative telephone interview: October 2019; E = Parent focus group alternative email interview: October 2019; Mother or Father followed by the number of the participant e.g. Mother 1

N.B. ‘Hospital’ is given as the admitting unit where no information was provided about the unit to which the child was admitted.

**Table 7 Stage1 Illness trajectories**

TH Teaching hospital, DGH District general hospital, CAU Child Assessment Unit, NP Nurse Practitioner

| **Parent identifiers** | **Age band of child** | **Duration of this illness pre-admission** | **Diagnosis for this illness** | **Illness trajectory** |
| --- | --- | --- | --- | --- |
| TH Mother A | 13-23 months | 3 + days | ?Bronchiolitis | Struggling with her breathing, rash as well, to GP Wednesday, sent to CAU, in CAU for 6 hours, doctors debated keeping her in, discharged home with leaflet ‘and told to look out for any recession’, Friday morning vomited after breakfast, struggling to breathe, called ambulance, admitted to HDU |
| TH Father B | Under 6 months | Approx. 7 days | RSV Bronchiolitis and Influenza A | Coughing for a week, choking during coughing bouts, visited GP three times, cough worsening and going blue for 5 days, then ED, no coughing during consultation so discharged home, ED again, coughing episode witnesses so sent to CAU, admitted to PICU (no timeframe information). |
| TH Mother C  Father D* | 2-4 year old | Approx. 6 days | ?Chest infection | Friday completed course of antibiotics, Mother away from home post surgery so cared for by Father (first time on his own), well until Sunday morning, Father detected high temp. gave Calprofen, called Mother, Mother visited Sunday evening, holds him, he is floppy, going grey around eyes and mouth, called ambulance Sunday evening, admitted to PICU. |
| TH  Mother E Father F | 2-4 year old | 1.5 days | ?Asthma attack and chest infection | Monday first ill, coughing and wheezing throughout the night, given inhalers, Mother didn’t want to wake Father so waited for surgery to open next day, Tuesday saw GP NP who gave nebuliser, called ambulance, admitted to PICU. |
| TH Mother G | 2-4 year old | Approx. 12 hours | Asthma attack and chest infection | Thursday morning high temp and slight wheeze, saw GP NP who advised ‘give him his pump’, more wheezy by midday so took him back to see NP early afternoon, told to carry on as before, by 5pm ‘gasping’ and pushing very hard to breathe whilst sleeping, waited for Father to come back from work, then to pack bags including food for Mother as it was Ramadan, picked up other children from after school club, taken to ED that evening by car, admitted to HDU |
| TH Mother H | 2-4 year old | 2.5 days | Chest infection and later pneumonia, fluid around the lung and Strep A blood infection | Family had all had ‘it’ in the preceding two weeks. Thursday first ill with temp, responsive to paracetamol, vomited in bed that evening, Friday slept on and off ‘really, really hot’, cared for by grandmother so Mother could Christmas shop, no bounce back on paracetamol, had wet herself when she woke, Grandmother advised seeking GP, Mother said she had but didn’t, Father went to work Christmas party and stayed at his parents’, Saturday morning lips ‘all white’, thought it was dehydration, called NHS111, ambulance sent, ED, ED consultant ‘on the fence’ about her until chest X-ray results, admitted to HDU/PICU |
| TH Mother L  Father M | 2-4 year old | 6 days | ADEM - Acute disseminated encephalomyelitis | Language difficulties. Sunday first ill with D&V and temp a bit high, Monday GP, Tuesday GP, told it was flu’, Wednesday ED with Father 6-7 hours told it was viral and sent home, getting worse and nose bleed, Thursday ED with teenage daughter to translate, taken less seriously than when Father took her so sent home, Friday not drinking or eating and floppy so evening to walk-in centre as it was close to them, took blood, told ‘low blood count’ sent to hospital ‘Just go now’, admitted to HDU/PICU. |
| TH Mother N  Father P* | 2-4 year old | 7 days | Tonsillitis with obstruction | Sunday cough, temperature responsive to paracetamol, walk-in centre red throat and given antibiotics, Wednesday no improvement > locum GP changed antibiotics, seemed to get a bit better until Saturday evening when she woke from sleep blue around lips and eyes, really struggling to breathe, called NHS111 who sent ambulance, resuscitated in ED, PICU |
| TH Mother Q Other carer R* | 6-12 month old | 10 days | Bronchiolitis (recurrence) with obstruction | Previous admissions with bronchiolitis, worse for him because he had tracheobronchomalacia. Worried about being judged by HCPs as paranoid parent. Friday first ill for this episode of illness. Much worse Wednesday and Thursday. Saturday seemed better. Late Sunday night/Monday morning Mother went to his room to find him really distressed, he gasped and stopped breathing. 1am Monday morning resuscitated at home by Mother, called ambulance, ED, PICU. |
| DGH Mother S Father T” | 13-23 months | 12 days | Collapsed lung and sepsis | Previous visits to ED with chickenpox, infection and high temp after immunisations. GP for antibiotics twice in preceding weeks, then Tuesday/Wednesday picked up a cold from playgroup, Wednesday following week GP tonsillitis and given antibiotics, felt reassured, Mother sent Father videos of him during the day, breathing quite hard, temperature hard to manage, relayed calling due to prior criticism from nurse, Friday night not eating or drinking or weeing so NHS 111 wanting OOHS GP, NHS 111 wanted to send ambulance but parents chose to take him in their care to ED, HDU |
| DGH Mother U Father V | 6-12 months | Approx. 8 days | Partially collapsed lung secondary to ?chest infection/pneumonia | A bit wheeze all week, then Monday a bit wheezy at nursery, Monday evening GP nothing to worry about, come back if it gets worse, Tuesday night woke from sleep really struggling, asked grandmother advised to seek help, sucking in at the ribs so called NHS 111 who sent ambulance, given nebuliser, taken to ED, HDU |
| DGH Mother W Father X | 2-4 year old | 7 days | Pneumonia | Monday sent home from nursery with temp., Tuesday GP to satisfy nursery, lots of people ill, reassured by having seen the GP, Saturday coughing at night, NHS 111 about midnight, Ambulance – sent away, Sunday phoned for appointment, GP appointment 2.30pm given antibiotics, evening not keeping fluids down, unable to stop coughing, called 999, advised to go to ED in their own car for speed, HDU/PICU |

*Lots of prior hospital admissions. “ Lots of prior visits to ED.

**Table 8 Stage 2 Illness trajectories**

FG = Parent Focus group T = Parent Focus group alternative telephone interview, E = Parent focus group alternative email interview, Mother or Father followed by the number of the participant e.g. Mother 1

| **Stage 2 Parent identifiers** | **Age band of child** | **Duration of this illness pre-admission** | **Diagnosis for this illness** | **Illness trajectory** |
| --- | --- | --- | --- | --- |
| Mother 1 FG | 6-12 months | Not known | Bronchiolitis | Previous experience of NHS 111 sending ambulance when it was not warranted put them off calling them and delayed help seeking. Mother’s Day, Mother out with friends, Father phoned to say breathing really bad, instructed Father to give inhaler, Mother came home and saw she was gasping for breath > to ED in their car > Adult resusc > Paediatric HDU |
| Mother 2 FG | 6-12 months | 4 days | Meningitis and sepsis | Bit of a temp for 4 days, gradually increasing > floppy, ‘*ash grey*’, tensing, vomiting, high temp. over 41 on paracetamol Friday night > Phoned NHS 111 (didn’t want to call 999 unnecessarily) > ambulance to ED 8pm at a weekend > ward at 1am for 27 hours > discharged but Mother refused to leave, Mother took photos to track visible changes in him and made notes > deteriorated, hand went black within 45 minutes > HDU > transferred to teaching hospital, legs black > right arm amputated, stroke. |
| Mother 3 FG | 2-4 year old | 3 days | Meningitis | Ill for 2 days in December, woke at midnight with high temp. unresponsive to paracetamol > ibuprofen, shaking > 6am whimpering, mottled skin, sunken eyes > watched TV, sore head > paracetamol worked > ate breakfast, napped, ‘*love bite*’ on his arm > glass test > checked symptoms on google >phoned GP who said ‘*you decide’* whether to call 999 > called 999 > collapsed > phone grandad while waiting > fast response car, semi-conscious, given ABs >hospital. |
| Mother 4 FG | 6-12 months | 24 hours | Meningococcal septicaemia | Woke crying, high temp., came down in response to paracetamol, diarrhoea, slept with Mother, woke in the morning with funny breathing, very still > rang GP, no urgent appointments >took child to GP demanding to be see > GP told them to go straight to ED > PICU |
| Mother 5 FG & Father 6 FG | Under 6 months | 24 hours | Late onset group B streptococcus meningitis | Had a cold > GP as not ‘quite herself’, Mother worked there and GP trusted her judgement and didn’t examine her > early hours of the morning Mother ‘*jolted awake*’ as she hadn’t woken for a feed, floppy > rang 999 > hospital > **died** |
| Mother 7 FG & Father 8 FG | 2-4 year old | < 24 hours | Meningitis B | Came home from nursery saying back hurts (there were lots of coughs and colds about), went to bed as normal, sick in the night, up with her 5.30am, ‘*bruise*’ on her eyebrow, vomiting, very quiet, bath, spot on leg, just lying there, ‘knew something bad was wrong’ > 999 > ED leg purple > PICU > **died** 13 days later |
| Mother 9 FG | 2-4 year old | 3 days | Meningitis | Ill for 2 days, had a nap on the sofa, tried to wake him, eyes not right ‘*It was like he wasn’t there behind his eyes*’ > neighbour for help > hospital, unconscious > resusc > **died** within a day. |
| Mother 10 FG | 2-4 year old | 24 hours | Meningococcal disease | Nursery Mon am, pm sofa day, then vomiting, rang GP – no appointments, high temp. in the evening, shaky and hallucinating, phoned 111 as husband thought need an ambulance, NHS 111 sent ambulance > ED, purple blotching on chest, rapidly spreading > ICU > transferred to London hospital |
| Mother 11 FG | 6-12 months | 2 weeks + | Pneumococcal meningitis | Ear infection, 3 lots of antibiotics, back to GP Friday 4pm, saw different doctor > ED Saturday as she was staring and stiff > Adult HDU > transferred to London hospital > brain dead Sunday > **died**. |
| Mother 12 FG | 6-12 months | 2 weeks + | Pneumococcal meningitis | Ill on and off for 2 weeks > walk-in centre > sent home, suddenly very, very sick at night, spine and head hurt > saw GP 9am, told ‘*nothing that sinister’* but Mother asked if he should go to ED, GP response ‘*I guess’* > ED, deteriorated within an hour > in hospital for 10 days. |
| Mother 13 T | 13-23 months | 2 days | Bacterial meningitis and septicaemia | Weekend. Woke in the night on Friday, vomited, high temp.. A bit unwell Saturday had a couple of spots > glass test, ‘*kind of disappeared’*, temp 39.7 > rang OOHS GP > saw GP almost immediately, temp over 40 >referred to hospital >discharged, told ‘*it’s probably just chickenpox’*, given advice sheet on caring for a child with a fever. Perked up, ate and drank, played with her sister. Vomited Saturday night, high temp.. Sunday morning floppy and not very responsive. Waited until Sunday early evening before taking her back to the hospital. Had a couple more spots. Admitted. Recorded diary of events during hospital stay. |
| Mother 14 T | Under 6 months | 12 hours | Viral meningitis | Bank holiday Monday. Day out on the beach. Irritable, thought it was the hot weather. On return home, sniffly and high temp. > checked NHS website >phone NHS 24 > OOHS Nurse Practitioner noticed distressed on handling and mottled legs> Ambulance > admitted.  Mother had no idea that it was serious. |
| Mother 15 T | Under 6 months | 12 hours | Meningitis | Grizzly and crying unusual for him one morning. Temp 38 > given paracetamol > temp continued to rise to 40, not feeding > asked grandmother, asked online groups, googled > rang GP > advised to ring 999 > grandmother drove them instead.  Had a ‘*small rash’*, blanched with glass test. Didn’t want to waste NHS time in an overburdened system. |
| Mother 16 T | Under 6 months | <24 hours | Meningitis and sepsis | Had gastroenteritis 10 days before. Wednesday poorly, crying on and off all day, overnight unsettled, feeding very little, large vomit after a feed, temp 39.2, grey/yellow colour > NHS 111 > OOHS appointment > phoned by Urgent care centre at hospital to come straight there instead, temp 39.9 & vomited > admitted. |
| Mother 17 E | Under 6 months | 6 days | Urinary sepsis | Initially snuffly on Wednesday/Thursday, Friday saw HV who noted she was unwell but not concerned, 11pm woke with temperature > Called NHS 24, ‘*just a cold’* > googled, read NICE guidelines, Saturday not feeding, temp. over 39, lack of urine > NHS 24 > OOHS GP, not concerned, Sunday temp spikes, fretful not feeding, Sunday night breathing fast, funny cry, Monday pm floppy and lethargic ‘*she looks like she is dead*’, almost grey, temp 41 > GP > hospital.  NB Delayed help seeking after Saturday consultation due to criticism, false reassurance ‘*It’s just a cold’*. |
| Mother 18 E | Under 6 months | <24 hours | Meningitis and septicaemia | Just after Christmas, snow. High temperature > phoned GP, advised to give paracetamol and ibuprofen, monitor for new symptoms/worsening, if yes, ring surgery. Middle of the night, strange whinge, diarrhoea and a purple mark on his belly>checked for symptoms of meningitis online >rang GP OOHS > cottage hospital in the snow, OA lips turning blue, pale, heavy breathing, given Abs, oxygen >called ambulance >hospital >retrieval unit>children’s hospital PICU.  NB ‘*Unable to word it out (meningitis) to my husband or anyone on the phone’* |
