# Supplementary material for: Navigating uncertain illness trajectories for young children with serious infectious illness: a modified grounded theory study
Source: BMC Health Serv Res. 2022 Aug 30;22:1103. doi: 10.1186/s12913-022-08420-5 (PMC9427158; doi:10.1186/s12913-022-08420-5)
Supplement: Supplementary file 3 — Additional file 3: S3 Fig. Navigating uncertain illness trajectories paper: Source of images and copyright information [file 12913_2022_8420_MOESM3_ESM.docx]

# S3 Fig Navigating uncertain illness trajectories paper: Source of images and copyright information

**999 Emergency image**


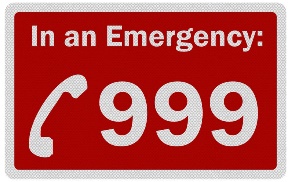


Located within Figure 5, manuscript page 27.

Source:

- https://www.google.co.uk/imgres?imgurl=https%3A%2F%2Ffirstaidforlife.org.uk%2Fwp-content%2Fuploads%2F2019%2F01%2F999-photo.jpeg&imgrefurl=https%3A%2F%2Ffirstaidforlife.org.uk%2Fdial-999%2F&tbnid=9V8j0XQQuTIarM&vet=12ahUKEwjn-5PBruLxAhUHPhoKHTPHAe0QMygAegUIARCCAQ..i&docid=JgH52YeiQGYZHM&w=1418&h=1000&q=999&hl=en-GB&ved=2ahUKEwjn-5PBruLxAhUHPhoKHTPHAe0QMygAegUIARCCAQ
- Google images links to https://firstaidforlife.org.uk/dial-999/ but that page does not show the above image so it has not been possible to contact the source or determine copyright status.
- Yahoo search has https://firstaidforlife.org.uk/wp-content/uploads/2019/01/999-photo.jpeg for the image but there is no copyright information on the page. Yahoo images also links the same image to https://zaharibb.com/2014/04/26/hello-999/ and https://www.newsx.com/about-us/ but the image is not on that page, and a cropped version of the same image on https://yourgrange.co.uk/when-to-call-999/ No copyright information or originator information on any of these pages.

**A&E Emergency Department Image**


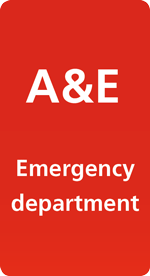


Located within Figure 5, manuscript page 27.

Source:

- Yahoo and Google images have the source as https://www.guysandstthomas.nhs.uk/our-services/emergency-care/accident-and-emergency.aspx but the image doesn’t appear on that page so no copyright information available.

**Call 111 image:**


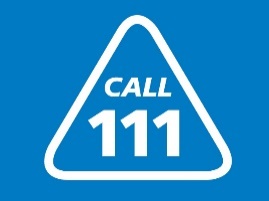


Located within Figure 5, manuscript page 27.

Source:

- Google has the source as https://www.northumberlandccg.nhs.uk/your-health/nhs-111/ but the image doesn’t appear on that page.
- Yahoo has the source as https://healthwatchderbyshire.co.uk/2021/01/think-you-need-to-go-to-ae-call-nhs-111-first/ but it isn’t on that page either.

If this image needs to be changed, the following is in the public domain (however the call 111 image is more appropriate for this manuscript):


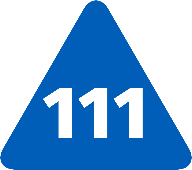


Source:

- https://en.wikipedia.org/wiki/NHS_111#/media/File:NHS_111_logo.svg
- This basic image is used in a wide range of forms (including the one we have used) in numerous places https://www.google.co.uk/search?q=Call%20111&tbm=isch&hl=en-GB&tbs&sa=X&ved=0CAEQpwVqFwoTCMDVw_ey4vECFQAAAAAdAAAAABAD&biw=1768&bih=863#imgrc=Xc73LSLBNTC8EM

**Home/family image:**


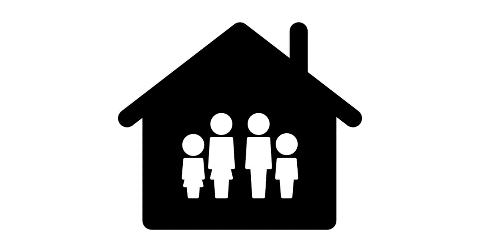


Located within Figure 5, manuscript page 27.

Source:

- Attribution ‘Family Home free vector icons designed by Freepik https://www.flaticon.com/free-icon/family-home_15767?k=1626260724243’

**Person/medical sign image:**


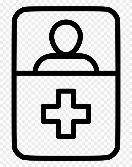


Located within Figure 5, manuscript page 27.

Source:

- https://www.clipartmax.com/middle/m2i8A0m2m2d3H7G6_patient-hospital-hospital-bed-medication-medicine-comments-icon/ ‘Free to use’ is the licence stated on the site.

**Primary Care image:**


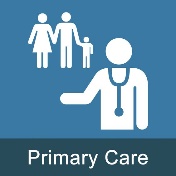


Located within Figure 5, manuscript page 27.

Source:

- Google has the source as https://twitter.com/ukpcrc which has a cropped version of the image as its logo – the website listed http://www.ukpcrc.co.uk/ is no longer valid. Their current website is https://ukpcrc.wordpress.com/ but the icon is not on the site.
- Yahoo has the source as http://inle.education/course/info.php?id=125 where it appears without attribution.
